# Parenting attitudes and pathological gaming: multifaceted influences of harsh-negative parenting on adolescent pathological gaming

**DOI:** 10.3389/fpsyg.2025.1521013

**Published:** 2025-06-06

**Authors:** Jae In Choi, Gyoung Mo Kim, Jeong Ae Kim, Eui Jun Jeong

**Affiliations:** ^1^Department of Digital Culture and Contents, Konkuk University, Seoul, Republic of Korea; ^2^Department of Humanities Counseling and Therapy, Konkuk University, Seoul, Republic of Korea

**Keywords:** pathological gaming, parenting attitude, harsh-negative parenting, social intelligence, adolescent gamer, aggression

## Abstract

**Introduction:**

As adolescent pathological gaming is increasingly recognized as a societal issue, previous research has aimed to identify the effects of family-related factors (e.g., parenting attitudes, parent–child relationship, etc.) and adolescents’ psychosocial factors (e.g., social skills, aggression, etc.). However, few studies have examined the associations among family-related factors, psychosocial factors, and pathological gaming simultaneously within a longitudinal research basis.

**Method:**

This study analyzed 3 years of longitudinal data collected from 968 adolescent gamers (*M* = 477, *F* = 491) in South Korea. A PLS-SEM method was employed using SmartPLS version 4 to reveal potential associations among parenting attitudes (harsh-negative parenting), psychosocial factors (social intelligence, aggression), and the degree of pathological gaming within a structural equation model.

**Results:**

The results indicate that harsh-negative parenting strongly influences social intelligence and aggression, potentially leading to pathological gaming. Specifically, harsh-negative parenting decreased the degree of social intelligence but also increased aggression. Social intelligence, in turn, was associated with a decrease in pathological gaming, while aggression was linked to an increase in pathological gaming.

**Discussion:**

These findings underscore the critical role and mechanism of parenting attitudes, which may influence adolescents’ pathological gaming through impacts on social intelligence and aggression. Adolescents’ psychological and social factors can be strongly affected by parents’ negative attitudes. In the context of preventing adolescent pathological gaming, more focus on policies or education aimed at parenting attitudes should be considered.

## 1 Introduction

In recent years, numerous factors associated with pathological gaming among adolescents have been investigated. The issue of persistent pathological gaming is significant enough to impede adolescents’ ability to function effectively in various domains, including personal, familial, social, educational, and occupational areas (Gonzalez-Bueso et al., 2020). While adolescents’ use of online games can be associated with positive effects such as stress relief (Barr and Copeland-Stewart, 2022), excessive gaming can be linked to negative outcomes, including reduced academic achievement (Toker and Baturay, 2016). This is corroborated by a recent meta-analysis on the factors related to pathological gaming (Düll et al., 2024).

With the World Health Organization declaring COVID-19 a pandemic in 2020 (World Health Organization, 2020), increased screen time has resulted in children spending more time on digital games (Susilowati et al., 2021). However, dedicating substantial time to gaming can lead to isolation from family and society. Previous research suggests that parental attitudes are among the factors influencing pathological gaming. For example, family relationships and conflicts are associated with internet gaming disorder (Bonnaire and Phan, 2017). Furthermore, excessive parental expectations have been directly correlated with problematic mobile game use in children (Jang and Ryu, 2016). Authoritarian parenting attitudes have also been significantly related to children’s tendencies toward pathological gaming (Akaroğlu, 2022).

Adolescents’ social intelligence is also influenced by parental attitudes. For instance, Kaur and Kalaramna (2004) argued that the home environment positively influences social intelligence. Parental warmth and involvement in education are positively associated with social intelligence, whereas excessive control is negatively associated with it (Nasir, 2015). Social intelligence is also a factor in internet addiction. A cross-sectional study involving 300 adolescents reported that a lack of social skills is associated with internet addiction (Chou et al., 2016). Meanwhile, aggression is a significant factor contributing to pathological gaming. Jeong et al. (2020) suggested that aggression accompanied by underlying dysfunctional salience and emotional regulation issues contributes to gaming disorder. Interestingly, children’s aggression has been closely related to parenting attitudes (Masud et al., 2019). Moudgil and Moudgil (2017) found a significant correlation between aggression and authoritarian mothers, and a significant negative correlation with permissive fathers. Additionally, uninformed, oppressive, and hostile parents, as well as single-parent or broken families, have been linked to increased screen time among adolescents (Kwon et al., 2011).

To understand adolescents’ behavior, it is important to consider a broader context rather than examining their identity and social relationships individually (Vorhies, 2009). While many researchers have studied how aggression can influence pathological gaming (Coyne et al., 2020; Kim et al., 2023; Krossbakken et al., 2018; Lemmens et al., 2011), the influence of negative parenting attitudes on adolescents’ social intelligence, and subsequently, how social intelligence may affect adolescents’ pathological gaming, has been studied only separately. Thus, despite the existence of potential predictors of pathological gaming related to familial environment and psychosocial factors, research considering these three factors together is scarce. Few studies have attempted to explore the mechanisms of pathological gaming from a multifaceted perspective, encompassing both familial and social aspects of adolescence. Therefore, this study aims to investigate the multifaceted mechanisms previously researched in the context of pathological gaming, focusing on the familial factor of harsh-negative parenting and the psychosocial factor of social intelligence. This study seeks to fill this gap.

## 2 Literature review and hypotheses development

*Pathological gaming* refers to a user’s loss of control over their gaming use and persistent, repetitive, and excessive engagement in gaming despite social and mental problems (Ferguson and Ceranoglu, 2014; Smith et al., 2015). It is generally recognized that pathological gaming is particularly threatening to adolescents who are familiar with gaming culture and heavy internet users (Kim and Kim, 2015), and it has been reported to lead to interpersonal and health problems, poor achievement, burnout, or poor psychological health (Nakayama et al., 2017; Zhuang et al., 2023). As pathological gaming has gradually emerged as a social problem, the Diagnostic and Statistical Manual of Mental Disorders (DSM-5) has included an IGD (Internet Gaming Disorder) category, and specific diagnostic criteria such as preoccupation, withdrawal, and tolerance have been discussed (Rehbein et al., 2015). In addition, various studies are being conducted to identify the harmful aspects and user characteristics in order to mitigate the harm caused by pathological gaming (Przybylski et al., 2017; Rafiemanesh et al., 2022). Notably, despite some controversy (Aarseth et al., 2017), the World Health Organization (WHO) has decided to list gaming disorder in the International Classification of Diseases (ICD-11) as World Health Organization (2018). Since then, attempts to explore protective and risk factors to prevent and minimize the harms of pathological gaming are increasingly being made.

In general, video gamers play games based on motivations such as fulfilling social interaction or competition needs and escaping from reality. Non-addicted or non-problematic players may share these motivations, whereas individuals exhibiting pathological gaming may engage in compulsive gameplay as a way to cope with their circumstances. This suggests that the association between gaming motivation and pathological gaming may not be particularly strong (Martucci et al., 2023). Excluding coping mechanisms specific to problematic players, non-problematic players tend to engage in gaming as a temporary escape from reality, as well as for social activities or a sense of achievement. While social aspects of gaming motivation reinforce these elements, when gaming motivation is driven by mood regulation or stress relief, gaming may also function as a coping mechanism for them (Gursesli et al., 2024a, 2024b).

In this regard, pathological gaming is not simply a result of heavy gaming use, but can be triggered by a complex interplay between sociosocial and individual psychological factors (Paulus et al., 2018). Therefore, when exploring the factors that lead to pathological gaming use, it is necessary to consider a wide range of individual psychological characteristics and environmental factors (Zhuang et al., 2023). Prior research suggests that pathological gaming is closely related to interpersonal and relationship dynamics, and can be influenced by family functioning and parent–child relationships (Bussone et al., 2020). In particular, given the nature of adolescents’ changing social relationships, parental nurturing attitudes are more important for adolescents with increased sensitivity to social stimuli (Orben et al., 2020). For example, a positive parental relationship has been identified as a key factor in securing adolescents’ psychological well-being and sense of social competence (Schacter and Margolin, 2019). On the other hand, negative parenting can lead to negative psychological problems in adolescents (Pinquart, 2017; Sukhodolsky et al., 2016), which in turn can lead to problematic behaviors such as pathological gaming use (Macur and Pontes, 2021; Paulus et al., 2018).

Negative parenting attitudes and the negative psychological issues reinforced by the attitudes can lead adolescents to become more overly engaged in gaming. This is also related to the medium characteristics of the game. For example, adolescents who suffer from negative parental relationships or a lack of social competence may find games more attractive, as they are more likely to vicariously satisfy their social deficits (Kardefelt-Winther, 2014; Kuss, 2013; Peeters et al., 2018). In addition, some adolescents may immerse themselves in virtual worlds to temporarily forget real-life psychological distress, such as relational problems (Bussone et al., 2020) or to release the urge to express accumulated anger (Lee et al., 2017). The present study examines how the adolescent-parent relationship affects psychological competence (social intelligence and aggression) and how it relates to pathological game use.

### 2.1 Negative parenting and pathological gaming

Positive parental relationships and parenting attitudes are essential for successful development during adolescence, when role and relationship transitions within the family occur (King and Delfabbro, 2017). Healthy parental relationships are known to support the development of psychological stability and social interaction in adolescents, when peer interactions become important (Schacter and Margolin, 2019), and parental parenting attitudes in particular have been reported to be a key factor in adolescent identity development (Ragelienė and Justickis, 2016). Parenting attitudes encompass behaviors towards a child that include both positive aspects, such as emotional support, as well as negative aspects, such as control and interference (Ragelienė and Justickis, 2016). Positive parenting attitudes not only support children’s healthy psychological development and social competence, but also act as a protective factor against problematic behaviors such as pathological gaming use (Li et al., 2018). Effective parental intervention and the improvement of interpersonal skills are crucial in promoting healthier gaming habits and reducing adolescents’ escapism (Commodari et al., 2024). A harmonious parent–child relationship plays a key role in promoting adolescents’ recognition and acceptance of active parental intervention, alleviating online risky behaviors (Lo Cricchio et al., 2022; Wartberg et al., 2015), and these findings are being strengthened (Liu et al., 2023).

On the other hand, negative parenting attitudes and relationships are known to delay children’s development of psychological or social competence or increase their vulnerability to problematic behaviors by interfering with parent–child emotional support and relationship building (Macur and Pontes, 2021; Pinquart, 2017; Sukhodolsky et al., 2016). Negative parenting attitudes include low levels of affection and coercive, dominant parenting styles (Segrin and Flora, 2019); over expectation, in which parents make unrealistic expectations and psychologically pressure their children based on their potential rather than actual abilities; and over interference, in which parents interfere without recognizing their children’s independence (Jang and Ryu, 2016). Previous studies have reported that adolescents exposed to negative parenting attitudes are more likely to develop negative psychological states such as increased aggression and lower social intelligence (Masud et al., 2019). Recent studies have also reported that parental intervention to protect adolescents from pathological gaming, when combined with psychological control, may be associated with the reinforcement of pathological gaming (Görgülü and Özer, 2024).

In other words, changes in adolescents’ psychosocial factors due to negative parenting attitudes may increase their vulnerability to pathological gaming use. In this context, negative parenting attitudes are known to be strongly associated with adolescent problem behaviors such as substance use (Paulus et al., 2018; Calafat et al., 2014).

According to prior studies, negative parenting attitude is a significant predictor of pathological gaming in adolescents (Chen et al., 2020). For example, excessive interference and control over their children may increase their desire to play games. If parents unreasonably restrict their children’s access to games, a vicious cycle of parent–child conflict, which in turn leads to pathological gaming can be made (Schneider et al., 2017). Parents’ excessive expectations, such as academic achievement, can also create excessive psychological pressure on their children, which can lead to psychological disempowerment and pathological gaming behavior (Jang and Ryu, 2016; Jeong et al., 2019). One study reported that negative parenting attitudes such as harsh control, psychological control, authoritarian, and permissive toward children were strongly associated with maladaptive behaviors such as externalizing problems (Pinquart, 2017). In addition, a study of 357 Chinese adolescents found that positive parenting attitudes, such as parental emotional support, were protective factors against pathological gaming, while negative attitudes were potential risk factors for pathological gaming (Chen et al., 2020). Similarly, a panel study of 1800 children and adolescents found that parental over-expectations were a predictor of pathological gaming use (Jang and Ryu, 2016). A meta-analysis examining the relationship between parenting attitudes and pathological gaming also found that coercive and interfering parenting attitudes were positively associated with pathological gaming (Li et al., 2018). In line with this empirical evidence, a growing body of research suggests that prevention of problematic gaming behavior should be based on family-based strategies that take into account parent–child relationships and parenting styles (King and Delfabbro, 2017; Wu et al., 2016).

### 2.2 Aggression, social intelligence and pathological gaming

Pathological gaming is strongly influenced by an individual’s psychological temperament. Aggression and social intelligence are the important temperamental factors that predict pathological gaming. In particular, negative psychosocial factors caused by parents’ negative parenting styles are likely to influence pathological gaming by increasing children’s vulnerability (Masud et al., 2019).

#### 2.2.1 Aggression and pathological gaming

Though recent meta-analysis and systematics studies of adolescent’s pathological gaming, many results shown that aggression is important factor associated with pathological gaming (Düll et al., 2024; Hammad and AL-shahrani, 2024; Limone et al., 2023; Li et al., 2023). Aggression refers to any behavior performed with the intention of causing harm to others. Aggression is strongly associated with social and mental health problems, including loneliness, depression, impulsivity, and emotion dysregulation (Chen et al., 2014; García-Sancho et al., 2014; Piko and Pinczés, 2014; Rothenberg et al., 2019) Aggression is also a risk factor for the formation of negative psychological states in individuals, as well as for bullying, cyberbullying which involve repeated acts of aggression against others, and problematic behaviors such as suicide and addiction (Lansford, 2018; McCloskey and Ammerman, 2018; Naaijen et al., 2020).

Despite the importance of peer relationships during adolescence, parenting styles still significantly impact on children’s personal and social development. Negative parenting attitudes and parent–child conflict are known to promote aggressive behaviors such as aggression or externalizing problems in children (Muñoz et al., 2017; Smokowski et al., 2015). In this regard, a longitudinal study of 447 adolescents over a three-year period reported that parental strict control was associated with increased aggression in children (Malonda et al., 2019). The study also found that positive parenting attitudes and support from parents had a significant effect on children’s prosocial behavior. Other studies have also shown that harsh and inconsistent parenting styles increase aggressive behavior in children (Sukhodolsky et al., 2016), and that authoritarian parenting styles, in which parents are highly directive and overly interfering with their children to make them obey their wishes, lead to increased aggression in children (Masud et al., 2019).

Aggressive users have higher pathological gaming scores than non-aggressive users (Lemmens et al., 2015). In a study of 263 Korean adolescents, both family conflict and aggression were significant predictors of pathological gaming, with aggression showing a mediating effect (Yuh, 2018). Other studies have also suggested the possibility of a positive association between pathological gaming and individual aggression, including hostility (Yen et al., 2017; Yen et al., 2018). In addition, pathological gamers are more highly correlated with the “competition” domain than those who do not (Rafiemanesh et al., 2022), it is suggest that users with high levels of aggression may become synchronized and over-engaged in game use to satisfy their need for aggressive behavior.

#### 2.2.2 Social intelligence and pathological gaming

Social intelligence refers to the ability to negotiate favorably in complex social relationships and conflicts by reading other people’s minds and understanding their intentions and motivations (Ganaie and Mudasir, 2015). In other words, social intelligence is the ability to understand and manage others, and then to recognize and respond to social situations properly (Komlósi, 2014; Holm et al., 2021). High social intelligence is associated with empathy, conflict management, organizational cooperation, and social pattern recognition (Segrin and Flora, 2019).

Previous research has shown that the development of social intelligence is also closely related to parenting attitudes. Positive parenting attitudes (e.g., parental acceptance, rational responsiveness, and affection for children) help adolescents internalize socially important values and become more socially mature (Segrin and Flora, 2019). On the other hand, negative parenting attitudes are known to limit children and adolescents’ opportunities for social maturation, which can lead to psychological problems such as decreased friendship and depression (King et al., 2016).

Social intelligence has also been related to the development of psychosocial characteristics in adolescents. In particular, the relationship between aggression and social intelligence has been the subject of conflicting research. Traditionally, high social intelligence has been thought to be a deterrent to aggression toward others, and it is believed that immaturity or deficits in social skills can be shown as negative behaviors such as bullying (Pabian and Vandebosch, 2016). Similarly, high levels of cognitive ability are known to contribute to emotional responses such as inhibiting impulsive emotions (Tang and Schmeichel, 2014). Empirical research has shown that people with sufficient self-esteem are less likely to verbally attack others online as their social skills grow (Savage and Tokunaga, 2017). On the other hand, some research suggests that not all aggressive behavior is due to a lack of social skills, and that there is a complex relationship between social intelligence and aggression. For example, high social intelligence may be associated with indirect aggression or relational aggression, analyzing social situations and using them to manipulate or hurt others, and sometimes pressuring others in one’s favor (Björkqvist, 2018; Loflin and Barry, 2016). However, there are also studies that show that social intelligence is unrelated to aggression (Dyches and Mayeux, 2015). These conflict results raise the need to reconfirm the relationship between aggression and social intelligence.

Social intelligence is one of the risk factors for problematic behaviors such as addiction. For example, Low levels of social intelligence are strongly associated with high levels of social anxiety, which can lead to pathological gaming among users who need to repair social relationships (Holm et al., 2021; Laconi et al., 2017; Von der Heiden et al., 2019). This is because people who struggle with social relationships in real life may over-immerse themselves in games to alleviate negative emotions and gain social support (Mun and Lee, 2022). On the other hand, high levels of sociability are known to be a strong protective factor against pathological gaming (You et al., 2017). In this regard, a study of 582 Korean middle and high school students found that those who engaged in pathological gaming had lower social intelligence and social capital scores than those who did not (Kim and Kim, 2023). In addition, a study of college students found that those who exhibited pathological gaming behavior had higher levels of interpersonal distortions and dysfunctional beliefs than those who did not (Çelik and Odacı, 2013).

### 2.3 Research model and hypotheses

**Figure 1:**
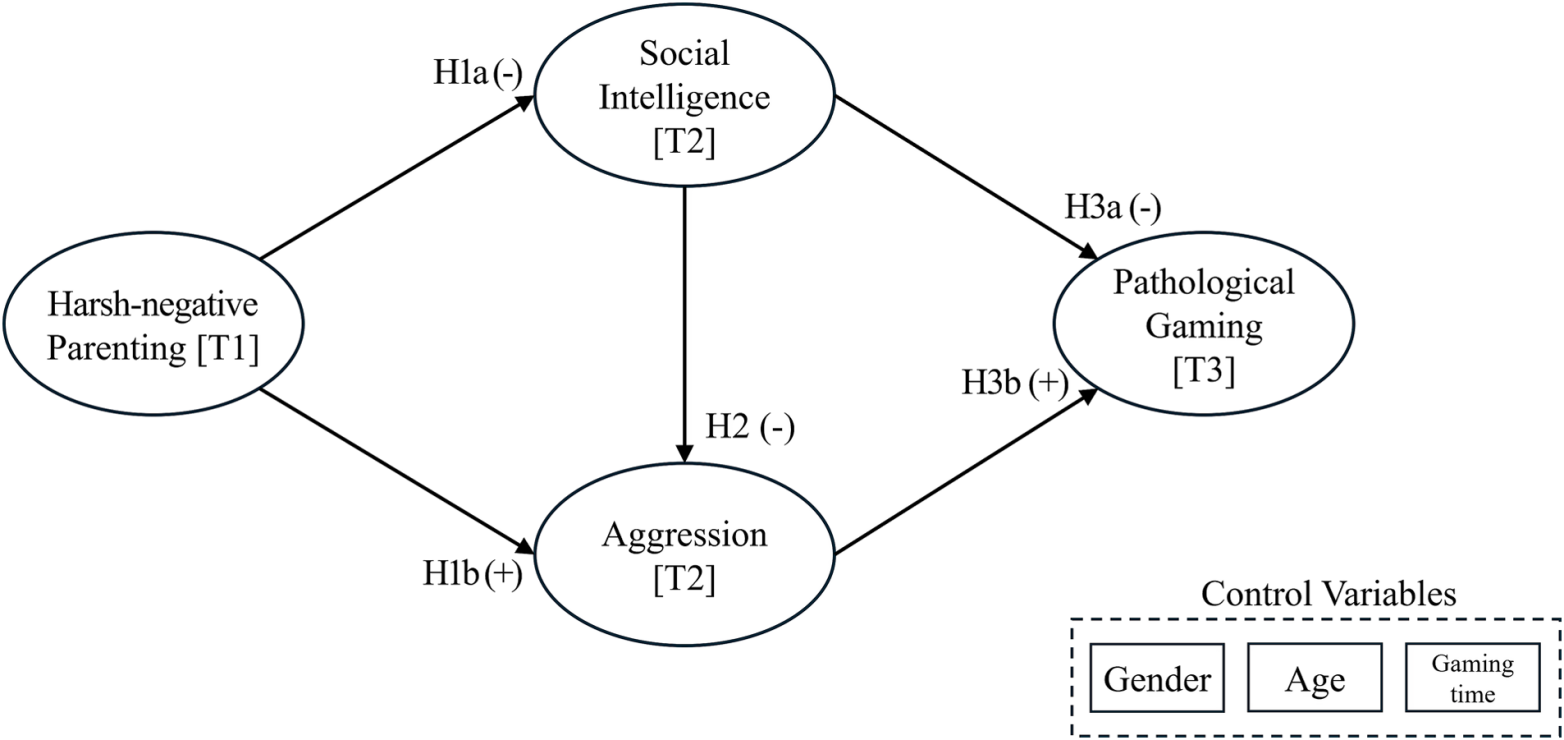
Research model.

This study examine the impact of harsh-negative parenting on the development of pathological gaming behavior in adolescents, this research investigates the potential consequences of parental over-interference and over-expectations on adolescents’ pathological gaming, considering their levels of aggression and social intelligence. The conceptual framework illustrated in Figure 1 indicates that harsh-negative parenting practices, which encompass both over-interference and over-expectation, may exert an influence on the social intelligence of children. Furthermore, harsh-negative parenting can be regarded as a contributing factor to heightened levels of aggression among adolescents. Also, social intelligence may influence aggression. Lastly children’s social intelligence and aggression can eventually lead to pathological gaming. Along with these factors, this study considered the adolescents’ sex, age, and average gaming time per day as control variables to find out if these factors influence pathological gaming.

*H1*. Harsh-negative parenting (HNP) is negatively associated with adolescents’ Social intelligence (SIT) (H1a), while Harsh-negative parenting is positively associated with adolescents’ aggression (AGR) (H1b).

*H2*. Social intelligence is negatively associated with adolescents’ aggression

*H3*. Adolescent’s social intelligence is negatively associated with pathological gaming (PTG) (H3a), while adolescent’s aggression is positively associated with pathological gaming (H3b).

## 3 Methods

### 3.1 Data collection

This study utilized panel data from the “Youth Game User Cohort Survey” conducted by the Korea Creative Content Agency (KOCCA) between 2015 and 2018 to evaluate the gaming behaviors of elementary, middle, and high school students in Korea. The data collection received prior approval from the Ethics Committee of Konkuk University, the collaborating institution. During the survey process, informed consent was obtained from respondents and parental approval was secured. Measures were implemented to protect personal information and ensure anonymity during data collection. A quota sampling approach based on school performance and gender balance was adopted. The data were collected through three face-to-face interview surveys conducted at one-year intervals by trained professionals following standardized survey protocols. The interviews adhered to established survey guidelines, and the same questionnaire was used throughout the study to maintain overall consistency. Consequently, participants received the same questionnaire for the duration of the study and were compensated USD 27.00 each. Comprehensive details on the survey methodology and dataset can be found on the Korea Creative Content Agency Game User Panel website (www.kocca.kr, accessed September 2023).

Of the 968 students surveyed for our analysis, 477 (49.3%) were male and 491 (50.7%) were female. As of their age, 345(35.6%) were attending elementary school, 333(34.4%) were attending middle school, and 290(30%) were attending high school. The daily gaming time was examined in 8 stages; Under 30 min, Less than 30 min to 1 h, Less than 1 to 2 h, Less than 2 to 3 h, Less than 3 to 4 h, Less than 4 to 5 h, Less than 5 to 6 h, Over 6 h. Table 1 summarizes the demographic characteristics of the study respondents.

**Table 1:** Demographic characteristics.

Characteristics		All participants (968)	
		Frequency	(%)
Gender	Male	477	49.3
	Female	491	50.7
Age group	Elementary group	345	35.6
	Middle school	333	34.4
	High school	290	30
Online game duration (Daily average)	Not playing	100	10.3
	Under 30 m	198	20.5
	30 m ~ 1H	213	22
	1H ~ 2H	205	21.2
	2H ~ 3H	134	13.8
	3H ~ 4H	68	7
	4H ~ 5H	22	2.3
	5H ~ 6H	10	1
	Over 6H	18	1.9

### 3.2 Measurement

A structural equation modeling (SEM) and repeated measures analysis, which uses the GLM (General Linear Model), were used to verify the research questions. Because this study is composed of dichotomous factors (social capital level: higher and lower), the repeated measures GLM can test the meaningful influence of social capital levels and change over time. It is also an appropriate method to measure whether time functions as a factor regulating social capital. This study utilized SEM to estimate and analyze the cause and effect between subject factors, or the entire panel. The GLM was supplemented with the SEM analysis, which increased the estimation efficiency that considers time-sequential characteristics. The SEM also presented extremely useful results for analyzing the correlations between factors from the perspective of the entire model.

*Harsh-negative parenting* was measured using a scale from the Heo (2013), which is based on the test developed by Huh (2004). This parenting attitude test was originally designed by Heo, but the questions regarding fathers and mothers were modified to include general parental questions, and redundant items were removed for the Youth Panel. The modified questions from the Youth Panel were used in this study (Jeon et al., 2021). The test consists of components such as parental supervision and affection, and for this research, the Over-Expectation and Over-Interference subscales were selectively used. The scale consists of eight items, each rated on a 4-point Likert scale from “1 = Not at all” to “4 = Very much.” Sample items from the scale include, “I always feel overwhelmed by my parents’ expectations, which I cannot fulfill,” and “My parents interfere by telling me what to do, even with small matters.”

*Social intelligence* was measured using the Tromso-Social intelligence scale (Silvera et al., 2001). The scale consists of 21 items, rated on a 7-point scale from “1 = not at all” to “7 = very much.” The subscales are social information processing, social skill, and social awareness, each consisting of seven items. Items such as “I can predict other people’s behavior,” “I adapt well to social situations,” and “I often hurt others without realizing it” are included in the scale.

*Aggression* was measured using the Short-Form Buss-Perry Aggression Questionnaire (BPAQ-SF). It was developed by Bryant and Smith (2001) as a shortened version of The Buss-Perry Aggression Questionnaire (BPAQ), a 29-item aggression scale designed by Buss and Perry (1992), which was modified by Diamond et al. (2005) to 12 items. It is rated on a 5-point scale ranging from 1 = not at all to 5 = very much. The subscales of the aggression questionnaire are categorized as Physical Aggression, Verbal Aggression, Anger, and Hostility. Example items from the aggression scale include “I often disagree with others,” “Sometimes I get angry suddenly and for no reason,” and “I have trouble controlling my anger.”

*Pathological gaming* was measured using the Internet Addiction Scale (Young, 2009), which was modified and adapted to the gaming context by incorporating questions specifically addressing gaming behaviors. It consists of 20 items and is rated on a 5-point scale ranging from “1 = not at all” to “5 = very much.” According to Widyanto and McMurran's (2004) item analysis of the IAT, it consists of salience, excess use, neglecting work, neglecting social life, lack of self-control, and anticipation. Survey questions include “I neglect other tasks because of gaming,” “I have problems in school because of gaming,” and “I feel bored and empty without gaming.”

*Gaming time* was measured as “average daily online gaming time” among players through 8 stage questions; Under 30 min, Less than 30 min to 1 h, Less than 1 to 2 h, Less than 2 to 3 h, Less than 3 to 4 h, Less than 4 to 5 h, Less than 5 to 6 h, Over 6 h.

## 4 Results

### 4.1 Reliability and validity test

We measured the levels of peer-perceived harsh-negative parenting (T1) among 968 adolescents and subsequently assessed their social intelligence (T2), aggression (T2), and pathological gaming (T3). There was a 1 year interval between each time point (T1, T2, and T3). Furthermore, we employed the Partial Least Squares Structural Equation Modeling (PLS-SEM) method for data analysis using SmartPLS version 4.0.9.6. PLS-SEM involves evaluating the measurement model using statistical criteria such as convergent validity (e.g., factor loading values and Average Variance Extracted or AVE), internal consistency reliability (e.g., Cronbach’s alpha and Composite Reliability or CR), and discriminant validity. To meet acceptable standards, we required individual item loading values to be at least 0.7 for better convergent validity and an AVE of at least 0.5. Internal consistency was assessed using Cronbach’s alpha and CR, with a minimum threshold of 0.7 for each construct (refer to Table 2). We also assessed discriminant validity using the heterotrait–monotrait (HTMT) ratio, with an acceptable threshold of up to 0.9 (refer to Table 3). Our analysis confirmed the presence of convergent validity, internal consistency reliability, and discriminant validity in the measurement model. Subsequently, we conducted data analysis using SmartPLS.

**Table 2:** Results for measurement model.

Scale/Items	M	SD	Cronbach’s α	CR	AVE	*R* ^2^
Harsh-negative parenting(HNP)/second-order						
Over-interference (OIN)	2.17	0.658	0.702	0.706	0.626	0.779
Over-expectation (OEX)	2.18	0.705	0.73	0.73	0.65	0.789
Pathological gaming (PTG)/first-order	1.94	0.854	0.94	0.943	0.95	0.236
Aggression (AGR)/first-order	1.76	0.789	0.871	0.876	0.907	0.327
Social intelligence (SIT)/first-order	1.94	0.854	0.88	0.889	0.909	0.014

M, Mean; SD, Standard Deviation; CR, Composite Reliability; AVE, Average Variance Extracted; R^2^, R Square Adjusted.

**Table 3:** Heterotrait-Monotrait Ratio (HTMT) for discriminant validity.

Variables	HNP	PTG	AGR	SIT
Harsh-negative parenting (HNP)				
Pathological gaming (PTG)	0.214			
Aggression (AGR)	0.251	0.368		
Social intelligence (SIT)	0.143	0.331	0.628	

Shaded boxes are the standard reporting format of PLS-SEM HTMT analysis.

### 4.2 Research model test

**Figure 2:**
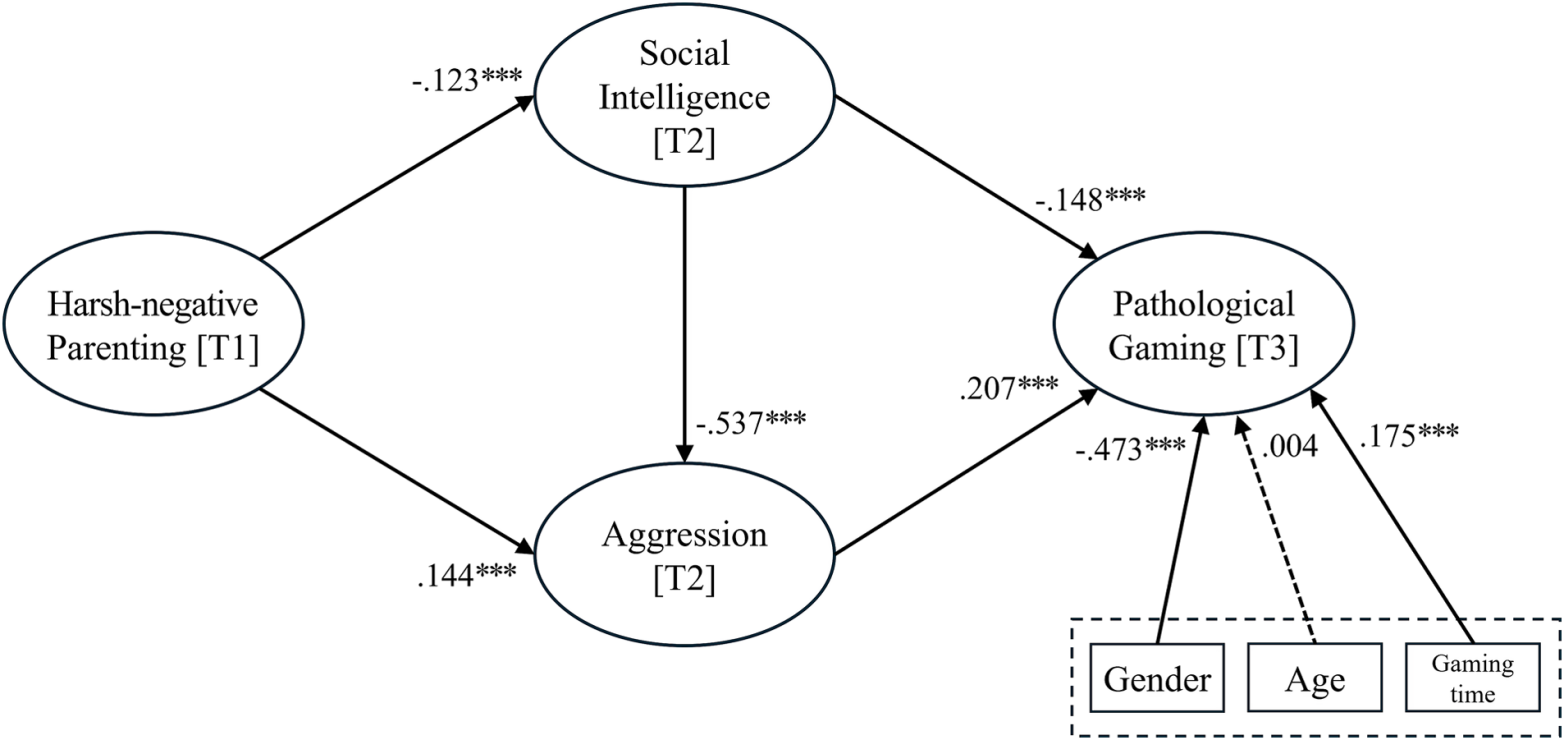
Results of the hypothesis tests.

For the research questions, structural equation analysis was conducted using SMART PLS. The results of hypothesis tests showed that all hypotheses are accepted (refer to Table 4). The results presented in Figure 2 showed that harsh-negative parenting had a negative effect on children’s social intelligence (*β* = −0.123, *p* < 0.001). However, harsh-negative parenting had a positive effect on children’s aggression (*β* = 0.144, *p* < 0.001). Social intelligence had a negative effect on aggression (*β* = −0.537, *p* < 0.001). Social intelligence had a negative effect on pathological gaming (*β* = −0.148, *p* < 0.001). On the other hand, aggression had a positive effect on pathological gaming (*β* = 0.207.001). The control variable, age had no significant effect on pathological gaming. However, gaming time had a positive effect on pathological gaming (*β* = 0.175, *p* < 0.001). Also, gender had a significant effect on pathological gaming (*β* = −0.473, *p* < 0.001). Male participants were more prone to pathological gaming, while female participants were less. This result is consistent with previous research indicating that male adolescents are more vulnerable to pathological gaming (Stevens et al., 2020; Paulus et al., 2018; André et al., 2022).

**Table 4:** Results of the hypothesis tests.

Hypothesis	Coef.	Mean	SD	T	Results
H1a. Harsh-negative parenting (HNP) → Social intelligence(SIT)	−0.123	−0.124	0.035	3.504***	Accepted
H1b. Harsh-negative parenting (HNP) → Aggression (AGR)	0.144	0.143	0.029	5.003***	Accepted
H2. Social intelligence (SIT) → Aggression (AGR)	−0.537	−0.537	0.024	22.501***	Accepted
H3a. Social intelligence (SIT) → Pathological gaming (PTG)	−0.148	−0.148	0.035	4.236***	Accepted
H3b. Aggression (AGR) → Pathological gaming (PTG)	0.207	0.207	0.037	5.657***	Accepted

Coef., Coefficient; Significant level: ***p < 0.001.

## 5 Discussion

### 5.1 Findings

This study examines whether harsh-negative parenting indirectly impacts pathological gaming through the relationships among key factors in our research model. Specifically, we investigate whether harsh-negative parenting, characterized by parental over-expectations and over-involvement, influences pathological gaming indirectly via its association with social intelligence and aggression.

Our study findings are as follows. Firstly, it was found that harsh-negative parenting could potentially exert a negative influence on adolescents’ social intelligence and may be associated with increased aggression. In exploring the relationship between harsh-negative parenting and pathological gaming, it was observed that harsh-negative parenting might have a stronger association with adolescents’ aggression, potentially diminishing their social intelligence. This suggests that familial factors could serve as influences on social dynamics and personality traits, as well as pathological gaming behaviors among adolescents.

Second, social intelligence, influenced by harsh-negative parenting, emerged as a significant factor affecting aggression, this is the most notable finding of the study. According to a meta-analysis on factors contributing to pathological gaming (Düll et al., 2024), aggression was identified as one of the influential factors associated with adolescents’ pathological gaming. In this context, social intelligence influenced by harsh-negative parenting may potentially contribute to aggression, emerging as a key finding of this study.

Therefore, an important consideration when analyzing our research model is the need to take into significant influenced and amount as between factors when examining the issue of adolescent pathological gaming. The findings suggest that social intelligence can be influenced by both aggression and harsh-negative parenting, indicating the necessity to view the phenomenon of adolescent pathological gaming in a broader context that encompasses environmental factors surrounding adolescents, rather than focusing solely on isolated variables.

### 5.2 Theoretical and practical implications

To the best of our knowledge, this study is among the early efforts to explore how parental parenting attitudes and adolescents’ social intelligence may be associated with aggression from the perspective of adolescent pathological gaming. Previous studies have independently explored the potential associations between harsh-negative parenting, social intelligence, and pathological gaming. However, these studies did not comprehensively address the complex interactions between these factors. To bridge this gap, our study aimed to examine whether harsh-negative parenting and adolescents’ social intelligence may lead to aggression, and how these factors may be associated with pathological gaming.

Our findings suggest that over expectation and over-interference, characteristics of harsh-negative parenting, may be associated with lower levels of social intelligence and higher levels of aggression, which, in turn, could contribute to pathological gaming. Both parental over-expectation and over-interference were linked to reduced social intelligence and elevated aggression, each further associated with increased levels of pathological gaming. Among the factors examined, aggression appeared to have the strongest association with adolescent pathological gaming. This aligns with previous research findings suggesting that aggression is associated with pathological gaming in adolescents (Gursesli et al., 2024a,b; García-Sancho et al., 2014; Kim et al., 2008; Griffiths et al., 2000). Additionally, harsh-negative parenting was found to have a somewhat stronger negative association with aggression than with social intelligence. These results indicate the potential influence of parenting attitudes—one of the family-related factors—on the development of pathological gaming in adolescents.

Furthermore, a key factor that warrants closer attention is adolescents’ social intelligence. According to our findings, the level of social intelligence was highlighted in the structural equation model as a significant determinant of aggression and was also associated with pathological gaming. The potential negative influence of social intelligence on aggression—possibly shaped by harsh-negative parenting and contributing to pathological gaming—suggests that fostering social intelligence may help mitigate pathological gaming. Our results further indicate that reduced social intelligence, stemming from parental over expectations and interference, may exert a greater influence on aggression than the direct effect of harsh-negative parenting on aggression. Parental over-involvement has been found to have a negative association with social intelligence (Ngai et al., 2018). Parenting style may influence adolescents’ social intelligence, influencing its development through parental practices and approaches. Just as adolescents develop social intelligence in response to these parenting behaviors, parents can likewise adapt their styles and practices to meet the evolving developmental needs of their children during adolescence (Segrin and Flora, 2019). This underscores the importance of addressing social intelligence when discussing the impact of parenting on adolescent aggression.

Our research findings confirm that adolescents’ social intelligence may influence pathological gaming and aggression. Adolescents with higher social intelligence are more likely to use social coping strategies in stressful situations due to a positive self-image (Maltese et al., 2012). On the other hand, adolescents with lower social intelligence may struggle with emotional regulation, leading them to choose negative coping strategies associated with pathological gaming (Lee et al., 2024). This suggests that social intelligence plays a significant role in how adolescents cope with problematic situations. Furthermore, social intelligence, within the context of pathological gaming, may exhibit more complex characteristics. Developing social intelligence is crucial for enhancing self-regulation and has been reported to complement the positive effects of personality traits such as agreeableness, openness, and extraversion (Kurmanova et al., 2024). This suggests a potential link between harsh-negative parenting and factors related to social intelligence. Research has demonstrated that parent-adolescent communication and social intelligence play a role in gaming behavior, including the association between violent game use and direct or indirect aggression (Wallenius et al., 2007). Therefore, regulating social intelligence could serve as one of the coping strategies to help manage adolescents’ problematic behaviors and aggression.

The finding that parenting attitudes may influence adolescents’ social intelligence, aggression and pathological gaming suggests that parents need to recognize the importance of their role in fostering an environment conducive to their children’s development as members of society. Aggression is known to be based on individual temperament, but research has long suggested that parenting styles can influence aggression as much as temperament (Olweus, 1980). Since the early stages of research on adolescents’ pathological gaming, aggression has consistently been reported as a significant psychological factor associated with this issue (Düll et al., 2024). This recognition refers to parents reflecting on whether their parenting practices have involved over-interference or expectations, as such practices can be relevant in addressing challenges like pathological gaming. Parents’ awareness of these issues can serve as a critical turning point in the development of preventive policies targeting pathological gaming, particularly those aimed at reducing aggression, and may offer a new framework for parental guidance in the digital era. In particular, caregiver education programs and the provision of guidelines for adolescents can help address the uncertainties caregivers may experience regarding online behavior management and setting standards for adolescents’ behavior in the rapidly evolving digital age. Additionally, school counseling or consultations with mental health professionals for adolescents who engage in digital behaviors, such as pathological gaming, as a coping strategy for harsh-negative parenting, as well as for their parents, may provide practical strategies for both parents and adolescents.

Over parental interference or expectations may negatively impact adolescents, with some children experiencing stress in the form of shame, helplessness, or worthlessness when they feel unable to meet their parents’ over-expectations (Deb et al., 2023; Wang et al., 2015). Today’s adolescents have faced unique developmental challenges, particularly during the pandemic, which has restricted their physical activity spaces and delayed opportunities for broader social participation. In an era where digital media use is inevitable, teaching children and adolescents how to engage with digital platforms, including gaming, in a healthy manner is essential for ensuring developmental opportunities (Paschke and Thomasius, 2024). Therefore, alternative parenting approaches beyond coercive control or intrusive interference in children’s gaming behaviors are essential for parents in the digital age. As an alternative to overbearing parenting, guidelines could focus on encouraging parents to adopt parenting attitudes that promote greater autonomy in their children’s everyday activities, including after-school, hobby, and leisure activities (Flamant et al., 2022). Ensuring children’s autonomy is necessary not only when they attempt new experiences but also when they face decisions involving unfamiliar outcomes.

However, this study has several limitations. First, the data were collected exclusively in South Korea, which limits the generalizability of the findings. Future research should aim to gather data from diverse cultural contexts to enable cross-country comparisons and improve the generalizability of the results. In the South Korean cultural context, online video gaming has become a prominent phenomenon, often equated with youth culture (Jin, 2010). Although the ultimate goals of “good parenting” may be consistent across cultures, the specific components that constitute good parenting practices can vary significantly depending on cultural norms (Vinden, 2001). Therefore, interpreting the present findings requires consideration of the broader cultural context—particularly differences in parenting practices and relatively low levels of gaming participation in some cultural settings. Future research may benefit from collecting data across diverse cultural contexts, which could allow for cross-national comparisons or culturally integrated analyses and ultimately enhance the generalizability of findings. Additionally, since the factors used in our study were measured through self-reports from the participants, which rely on the participants’ own perceptions, there may be slight variations when considering the potential for recall bias, social desirability bias, and common method variance. Therefore, to address limitations such as recall bias, future research may benefit from incorporating alternative methods, such as observing adolescents’ online gaming behaviors in naturalistic settings and conducting interviews with them (Mun, 2024). Furthermore, given that our data encompass adolescents from elementary to high school, it is limited in that there may be differences according to developmental stages within adolescence. Future research should consider including school level as a control variable or encourage the analysis of developmental stage differences within the same research model. Second, although this study focused on parenting attitudes, adolescents’ aggression, and social intelligence, it could have included additional variables related to pathological gaming. For example, the quality of social relationships outside the family, such as those with peers or teachers, plays an essential role during adolescence. Furthermore, other psychological factors, such as negative emotions, self-efficacy, and anxiety, may be relevant. Future studies should take a broader approach by examining not only the quality of adolescents’ social relationships but also the interplay of various parenting attitudes, aggression, and social intelligence to enhance the understanding of pathological gaming behavior. Therefore, the need for an integrated approach that dismantles traditional boundaries is emphasized by investigating not only parenting attitudes but also the interactions with other intrinsic and extrinsic factors, such as peer relationships, communication within social contexts, and aggression, which can significantly shape adolescents’ development.

## 6 Conclusion

This study suggests that harsh-negative parenting may be a significant factor associated with adolescent pathological gaming. The finding that harsh-negative parenting can be associated with adolescents’ psychosocial factors, aggression, and pathological gaming underscores a key implication of this research. When parents seek to address or prevent pathological gaming in adolescents, focusing narrowly on individual factors may be less effective. Instead, adopting a broader perspective that integrates family-related and psychosocial factors may provide more meaningful insights. Adopting a holistic approach to understanding these interconnected influences may offer more nuanced insights and prove more effective in informing targeted interventions.

## Data Availability

The datasets presented in this study can be found in online repositories. The names of the repository/repositories and accession number(s) can be found at: https://www.kocca.kr/gameguide/subPage.do?menuNo=203709. Further inquiries can be directed to the corresponding author.
